# Trends and factors associated with acute respiratory infection among under five children in Zambia: evidence from Zambia’s demographic and health surveys (1996-2014)

**DOI:** 10.11604/pamj.2020.36.197.18799

**Published:** 2020-07-20

**Authors:** Nelia Langa Mulambya, Francis Hamaimbo Nanzaluka, Ntazana Nana Sinyangwe, Mpundu Makasa

**Affiliations:** 1 Zambia Field Epidemiology Training Program, Lusaka, Zambia,; 2 School of Public Health, University of Zambia, Lusaka, Zambia,; 3 Zambia National Public Health Institute, Lusaka, Zambia,; 4 Ministry of Health, National Tuberculosis and Leprosy Program, Lusaka, Zambia

**Keywords:** Acute respiratory infection, children, under-five underweight trends, Zambia

## Abstract

**Introduction:**

acute respiratory infection (ARI) is one of the leading causes of morbidity and mortality among children under the age of five years globally accounting for 16% of deaths. In Zambia, ARI accounts for 30-40% of children's outpatient attendance and 20-30% of hospital admissions. We assessed trends and factors associated with ARI among under-five children in Zambia from 1996 to 2014.

**Methods:**

we analysed the Zambia demographic and health survey data for 1996, 2002, 2007 and 2014 of under five children and their mothers. We extracted data using a data extraction tool from the women's file. We analysed trends using chi square for trends. We conducted a complex survey multivariable logistic regression analysis, reported adjusted odds ratios (AOR) 95% confidence intervals (CI) and p-values.

**Results:**

we included a total of 6,854 and 2,389 (8%) had symptoms consistent with ARI. A 2% upward trend was noted between the 1996 and 2002 surveys but a sharp decline of 10% occurred in 2007. The chi2 trend test was significant p < 0.001. Children whose mothers had secondary or higher education were less likely to have ARI (AOR 0.30 95% CI 0.15-0.58) compared to those with no education. Underweight children had 1.50 times increased odds of having ARI (AOR 1.50 95% CI 1.25 - 1.68) compared with children who were not. Use of biomass fuels such as charcoal (AOR 2.67 95% CI 2.09 - 3.42) and wood (2.79 95% CI 2.45 -3.19) were associated with high odds for ARI compared to electricity.

**Conclusion:**

the prevalence of ARI has declined in Zambia from 1996 to 2014. Factors associated with occurrence of ARI included being a child under one year, underweight, use of biomass fuel such as charcoal and wood. Interventions to reduce the burden of ARI should be targeted at scaling up nutrition programs, as well as promoting use of cleaner fuels.

## Introduction

Acute respiratory infection is an acute disease affecting the lungs causing fluid and inflammatory waste to accumulate covering alveolar, inhibiting gaseous exchange and leading to difficult and painful breathing [1]. ARI affects all ages but the effects are particularly life threating among children under the age of five [2, 3]. Globally it is a leading cause of morbidity and mortality among under five children, in 2015, 922,000 deaths occurred accounting for 16% of all deaths in this age group [1, 4]. Low and middle income countries (LMIC) in sub-Saharan Africa and southern Asia account for half a million deaths [4, 5]. It is further estimated that six out of ten deaths among children are due to ARI. More than 160 million children develop ARI each year, 20 million get hospitalized and 1 million die [4]. WHO estimates that in developing countries, 40-60% of out-patients attendance and 30-40% of hospitalizations among children are due to ARI [1]. In Zambia, acute lower respiratory infections have remained among the major causes of morbidity and mortality.

The health management information system (HMIS) report of 2016-2017 shows ARI among the top ten causes of morbidity among under five children accounting [6] for 14% of deaths after malaria and diarrhoea [6, 7]. Thirty to forty percent (30 - 40%) of children´s out-patient attendance as well as 20-30% of hospitalizations in Zambia are due to acute respiratory infection [2]. Several factors are associated with ARI. These include age, residence and environmental factors such as, exposure to infectious diseases, malnutrition, poor hygiene and sanitation and biomass fuels and unhealthy environments [5, 8-10]. Children living in developing countries in remote regions where poverty, hunger and poor access to healthcare exist have increased risk of developing acute respiratory infections [8]. Little is known about the trends and the influence of sociodemographic, socioeconomic and environmental factors associated on ARI among under five children at country level. We sought to assess the trends of ARI across the four Zambia demographic and health survey (ZDHS) surveys and investigate sociodemographic and environmental factors associated with ARI among under five children in Zambia.

## Methods

We conducted a cross-sectional study using nationally representative Zambia demographic and health surveys of 1996, 2002, 2007 and 2014. Sociodemographic, socioeconomic and environmental data from the women´s file was extracted for analysis. Details of the ZDHS design are described elsewhere [11, 12]. Sociodemographic variables included mother´s age, education level, residence, child´s age, sex, vaccination, breast feeding, vitamin A in the last 6 months and anthropometric measurements using WHO definition. Others included employment status and wealth index were also extracted, environmental variables such as household overcrowding (based on Zambia public health act) and type of cooking fuel. We defined the dependent variable ARI as occurrence of cough accompanied by short rapid breaths that was chest related occurring two weeks preceding the survey. This was a binary variable coded as 1 if child had ARI and 0 otherwise. We analysed data using statistical software, STATA version 14.0 SE (STATA corporation college station, Texas).

A weighted survey analysis was done and a design effect of 1.490 [11] was used. Cross-tabulations and Pearson´s Chi-square tests were done to examine associations of study variables and p-values of < 0.05 were considered significant. We analysed trends using Chi-square tests for trend based on the prevalence of ARI across the datasets. We conducted bivariate logistic regression analysis and multivariable logistic regression analysis to control for confounding. P-value < 0.05 were considered significant at 95% confidence level (CI). We reported unadjusted and adjusted odds ratios (AOR) to measure the strength of association. We used the F-test to determine the best fit model. Ethical approval was obtained from excellence in research ethics and science ethics committee and the national health research authority and permission to use the ZDHS data sets was obtained from the Zambia central statistical office and measure DHS website. No harm was paused to the participants since the study utilized secondary data which was already de-anonymized.

## Results

**Figure 1:**
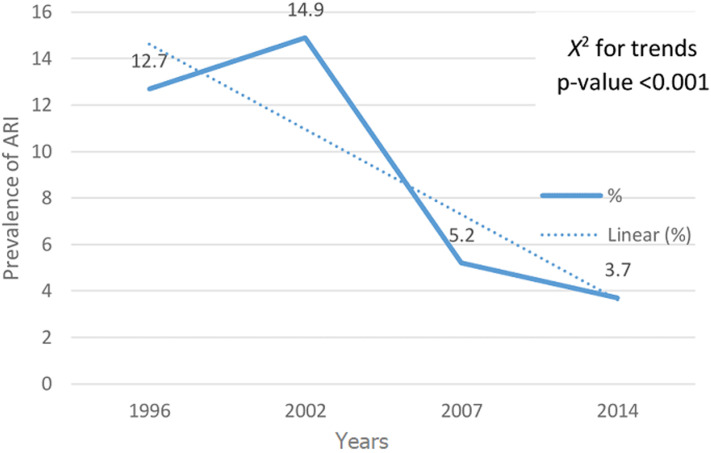
ARI trends among under five children in Zambia 1996-2014

**Figure 2:**
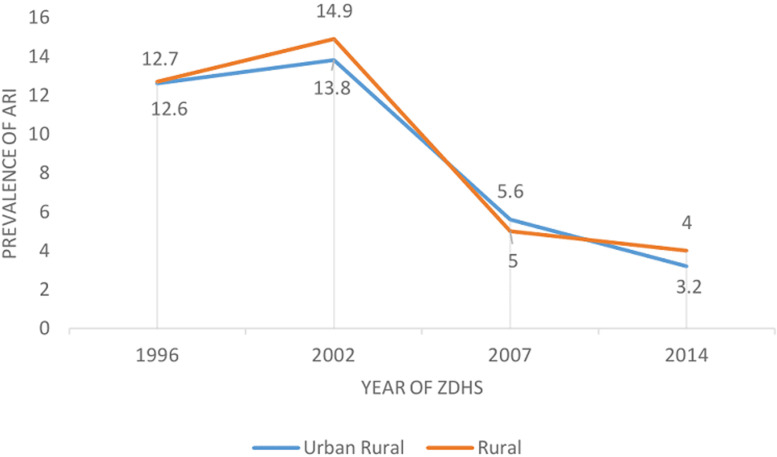
ARI trends among under five children by urban rural residence in Zambia

A total of 30,391 children under 5 years were analysed from 1996, 2002, 2007 and 2014 ZDHSs. A total of 6,854 (23%) children were reported to have had cough two weeks preceding the survey. Out of these, only 2,389 (8%) had symptoms consistent with ARI. Of these 50.2% were females and majority 21,274 (70%) lived in rural areas. The mean age of the children was 28 months. Majority 22,185 (73%) of the mothers were aged between 20-35 years and more than half 16,715 (55%) were working. The most common type of cooking fuel was wood 13,979 (46%) and only 19% of the households had electricity (Table 1, Table 2). Based on the trend analysis from 1996 to 2014, a 3% increase in prevalence was noted from 1996 to 2002 when the highest prevalence of ARI was recorded at 15%. The prevalence of ARI decreased drastically from 15% in 2002 to 5% in 2007. Chi square for trend was significant at p-value < 0.001 (Figure 1). The prevalence of ARI was higher in rural areas across the surveys except 2007 which showed high prevalence in the urban areas (Figure 2).

**Table 1:** frequency distribution of background characteristics of under five children with ARI in Zambia

Variable	1996 n (%)		p-value	2002 n (%)		p-value	2007 n (%)		p-value	2014 n (%)		p-value
ARI	No	Yes		No	Yes		No	Yes		No	Yes	
**Mothers Age**												
15-19	396 (7)	73(1)	0.3584 χ 2	480(8)	89(2)	0.8237χ2	337(6)	20(0.3)	0.8784χ2	833(14)	49(0.8)	0.2228χ2
20-24	1609 (26)	254(4)		1721(30)	262(5)		1410(24)	72(53)		2910(50)	107(2)	
25-29	1329 (22)	175(3)		1519(26)	202(3)		1579(27)	97(2)		3159(54)	121(2)	
30-34	1022 (17)	142(2)		982(17)	139(2)		1155(20)	62(1)		2568(44)	87(1.5)	
35-39	622 (10)	79(1)		660(11)	89(2)		659(11)	35(0.6)		1672(29)	68(1.2)	
40+	358 (6)	50(0.8)		425(7)	62(1)		418(7)	19(0.3)		1023(17)	35(0.6)	
**Mother Currently Working**												
No	2463(40)	361(6)	0.0475 χ 2	2084(36)	290(5)	0.0471χ2	2817(48)	111(2)	0.0014χ2	5448(43)	170(1)	0.0169χ2
Yes	2875(47)	410(7)		2856(49)	553(10)		2740	193(3)		6673(53)	299(2)	
**Residence**												
Urban	2084(34)	309(5)	0.0552 χ 2	1548(27)	248(4)	0.8237χ2	1602(28)	95(2)	0.5840χ2	4182(33)	136(1)	
Rural	3200(52)	464(8)		3396(59)	595(10)		3955(68)	209(4)		7983(63)	322(3)	
**Education**												
No education	719(12)	110(2)	0.0475 χ 2	689(12)	153(3)	0.0002χ2	755(13)	41(1)	0.5260χ2	1331(11)	56(0.4)	0.4562χ2
Primary	3429(56)	468(8)		3121(54)	535(9)		3507(60)	205(3)		6831(54)	260(2)	
Secondary	1091(18)	172(3)		1049(18)	149(3)		1164((20)	53(1)		3548(28)	144(1)	
Higher	110(2)	8(0.13)		87(2)	5(0.1)		131(2)	5(0.1)		444(4)	8(0.1)	
No education	719(12)	110(2)		689(12)	153(3)		755(13)	41(1)		1331(11)	56(0.4)	
**Sex**												
Male	2625	364(6)	0.7688χ2	2448(42.3)	428(7.4)	0.5487 χ2	2714(46)	173(3)	0.0080χ2	6163(49)	231(2)	0.5945χ2
Female	2711	409(7)		2496(43.1)	415(7.2)		2843(48.5)	131(2.2)		6002(48)	238(2)	
**Childs age**												
0-11	1107(18)	213(3.5)	0.0811χ2	1004 (17)	234(4)	0.0001 χ2	1203(21)	85(1.5)	0.0148χ2	2385(19)	93(0.7)	0.0153χ2
12-23	1144(19)	203(3.3)		1065 (18)	234(4)		1206(21)	66(1.1)		2450	126(1)	
24-35	1087(18)	152(2.5)		966 (17)	177(3)		1084(18)	67(1.1)		2450(19)	97(1)	
36-47	995(16)	117(2)		904 (16)	99(2)		1033(17.6)	44(0.8)		2368(19)	78(0.6)	
**Vaccination status**												
Incomplete	243(13)	27(2)	0.7116χ2	336(18)	41(2)	0.72 χ2	471	28	0.3278χ2	536(14)	16(0.4)	0.4031χ2
Complete	1411(77)	149(8)		1304(69)	206(11)		1262	56		3050(82)	103(3)	
**Breast feeding**												
No	2030(33)	251(4)	0.0115χ2	1798(31)	274(5)	0.1011 χ2	2042	105	0.5016χ2	5423(43)	206(1)	0.8443χ2
Yes	3306(54)	522(9)		3146(54)	569(10)		3515	199		6747(53)	262(2)	
**Under weight**												
No	4234(69)	598(10)	0.6221χ2	3736(65)	581(10)	0.006 χ2	4864(83)	261(1)	0.3968χ2	10583(84)	391(3)	0.0742χ2
Yes	1102(18)	175(3)		1208(20)	262(5)		693(12)	43(0.7)		1582(13)	77(1)	
**Vitamin A last 6months**												
No	na	na		1833(32)	338(6)	0.0185 χ2	739(22)	38(1)	0.5160χ2	3887(31)	115(1)	0.0052χ2
Yes	na	na		3007(52)	503(9)		2420(73)	133(4)		8278(66)	354(3)	

**Table 2:** frequency distribution of housing characteristics of under-five children with ARI in Zambia

Variable	1996 n (%)		p-value	2002 n (%)		p-value	2007 n (%)		p-value	2014 n (%)		p-value
ARI	No	Yes		No	Yes		No	Yes		No	Yes	
**Electricity**												
No	4256(70)	629(10)	0.0275χ2	3985(69)	728(13)	0.0013χ2	4782(82)	254(4)	0.9919χ2	9442(75)	364(3)	0.7651χ2
Yes	1080(18)	144(2)		836(14)	115(2)		775(13)	50(1)		2723(22)	105(0.8)	
**Main Floor Material**												
Earth	3,471 (56)		0.0744χ2	3189(55)	589(10)	<0.0003χ2	3749(64)	201(3.4)	0.8246χ2	7494(60)	306(2.4)	0.4107χ2
Ceramic/Polished wood	84 (1)			18 (0.3)	0		163(3)	0		97 (1)	0	
Cement	2,515(41)			1491(26)	216(4)		1634(28)	103(1.8)		4114(34)	163(1.3)	
Carpet	8 (0.1)			110(2)	34(1)		11(0.2)	0		53(0.4)	0	
**Cooking Fuel**												
Electricity	na		na	635(11)	74(1)	0.045χ2	585(10)	37(0.6)	0.7729χ2	972(8)	23(0.2)	0.0777χ2
Charcoal	na		na	1301(23)	216(4)		1289(22)	85(1.5)		4100(32)	145(1.2)	
Wood	na		na	3021(52)	537(9)		3683(63)	182(3)		7091(56)	301(2.4)	
Electricity	na		na	635(11)	74(1)	0.045χ2	585(10)	37(0.6)	0.7729χ2	972(8)	23(0.2)	0.0777χ2
**Household Size**												
Not crowded	2563(42)	369(6)	0.3994x2	2706(47)	486(8)	0.2515χ2	3353(57)	184(3)	0.9402 χ2	4550(36)	295(2.3)	0.6598χ2
crowded	2773(45)	404(7)		2238(4)	357(6)		2203(38)	120(2)		7615(60)	174(1.4)	

At bivariate analysis, an increase in mother´s age and higher level of education were associated with a decreased chance for ARI (odds ratio [OR] 0.63 95% confidence interval [CI] 0.50 - 0.80 P-value [p-] < 0.001) (OR 0.27 95% CI 0.17 - 0.40 p -< 0.001) compared with young mothers and those with no education (Table 3). Breast feeding children were more likely to suffer from ARI (OR1.94 95% CI 1.73-2.17 p -< 0.001) compared to those that were not. Lack of vitamin A supplementation in the last six months was 2.48 times associated with likelihood for ARI (OR 2.48 95% CI 2.22-2.77) (Table 3). Being in a crowded home was associated with increased odds for ARI (OR 1.06 95% CI 1.04-1.08 p < 0.001). The number of children five years and under in the household showed an increased chance of ARI (OR 1.10 95% CI 1.03-1.16 p-0.002). Using charcoal (OR 3.24 95% CI 1.73-2.17 p -< 0.001) for cooking had an increased odds for ARI compared to electricity (Table 3).

**Table 3:** predictor’s variables of ari among under-five children in Zambia

Variable	Odds Ratio (95%CI^*^)	P-value
**Mother's Age**		
15-19	1	
20-29	0.83 (0.69 - 1.04)	
30-49	0.90 (0.70 - 1.17)	
**Residence**		
Urban	1	
Rural	1.04 (0.95, 1.14)	0.372
**Level of education**		
No education	1	
Primary	0.71 (0.58 - 0.88)	
Secondary	0.66 (0.51 - 0.59)	<0.001
Higher	0.27 (0.13 - 0.84)	<0.001
**Childs age in months**		
0-11	1	
12-23	0.98 (0.87 - 1.11)	0.783
24-35	0.83 (0.73 - 0.94)	0.004
36-47	0.60 (0.52 - 0.69)	<0.001
**Sex**		
Male	1	
Female	1.00 (0.92 - 1.10)	0.887
**Vaccination Status**		
No	1	
Yes	1.03 (0.80 - 1.24)	0.772
**Under Weight**		
No	1	
Yes	1.94 (1.75 - 2.15)	<0.001
**Breast Feeding**		
Yes	1	
No	0.72 (0.65 - 0.80)	<0.001
**Vitamin A last 6 months**		
No	1	
Yes	0.44 (0.39 - 0.49)	<0.001
**Over Crowding**		
Not crowded	1	
Overcrowded	1.24 (1.13 -1.36)	<0.001
**Type of cooking fuel**		
Electricity	1	
Charcoal	3.24 (2.89 - 3.42)	<0.001
Wood	1.69 (2.45 - 2.07)	<0.001
**Electricity**		
No	1	
Yes	0.77 (0.64, 0.90)	0.001

**Multivariable analysis of factors associated with ARI among under five children:** at multivariable logistic regression, children whose mothers´ were older than 20 years had a reduced odds of having ARI (AOR 0.68 95% CI 0.52-0.89 p- 0.005) as compared to mothers younger than 20 years after controlling for education level, underweight, child´s age, vitamin A and overcrowding. Children whose mothers had higher education were 74% less likely (AOR 0.30 95% CI 0.15-0.58 p -< 0.001) odds of ARI. Children who were underweight had 1.50 times the odds of ARI compared to those who were not (AOR 1.50 95% CI 1.28 -1.68 p -< 0.001). Using charcoal for cooking was associated with 2.67 times increased odds of ARI (AOR 2.67 95% CI 2.09-3.4 p-<0.001) as compared to using electricity (Table 4).

**Table 4:** multivariable analysis of factors associated with ARI among under-five children in Zambia

Variable	Unadjusted odds ratios	p-Value	Adjusted Odds Ratio [95% conf. interval]	p-Value
**Mother's Age**				
15-19	1		1	
20-24	0.83 (0.70 -0.97)	0.018	0.77 (0.59 -1.00)	0.053
25-29	0.67 (0.57 -0.79)	<0.001	0.68 (0.52 - 0.89)	0.005
30-34	0.63 (0.53 -0.75)	<0.001	0.59 (0.44 -0.78)	<0.001
35-39	0.64 (0.53 -0.77)	<0.001	0.62 (0.46 -0.85)	0.003
40-49	0.64 (0.51 -0.78)	<0.001	0.61 (0.43 -0.86)	0.004
**Education**				
No education	1		1	
Primary	0.82 (0.73 -0.93)	0.002	0.72 (0.55 -0.95)	0.018
Secondary	0.66 (0.57 -0.76)	<0.001	0.64 (0.52 -0.80)	0.011
Higher	0.26 (0.17 -0.40)	<0.001	0.30 (0.10 -0.87)	0.026
**Childs age in months**				
0-11	1		1	
12-23	0.98 (0.87 -1.11)	0.783	0.84 (0.67 -1.07)	0.163
24-35	0.83 (0.73 -0.94)	0.004	0.85 (0.79 -1.19)	0.19
36-47	0.60 (0.52-0.69)	<0.001	0.58 (0.43 -0.78)	0.001
48-59	0.52 (0.45 -0.60)	<0.001	0.56 (0.41 -0.77)	<0.001
**Vitamin A last 6months**				
No vitamin	1		1	
Yes last 6months	0.44 (0.39 -0.49)	<0.001	0.84(0.68 -1.04)	0.111
**Underweight**				
No	1		1	
Yes	1.94 (1.75 -2.15)	<0.001	1.31 (1.10 -1.62)	0.015
**Household characteristics**				
Overcrowding				
Not crowded	1		1	
Crowded	1.15 (0.99 -1.33)	0.051	1.09 (0.87 -1.36)	0.441
**Type of Cooking fuel**				
Electricity	1			
Charcoal	3.24 (2.89 -3.42)	<0.001	2.67 (2.09 - 3.42)	<0.001
Wood	1.69 (1.39 -2.07)	<0.001	2.79 (2.45 - 3.19)	<0.001

## Discussion

The main aim of the study was to assess the trends of ARI and determine factors associated with ARI in Zambia. In the current analysis, age of child, underweight, mothers´ education level, vitamin A and type of cooking fuel were among factors associated with ARI. Findings from our study showed a reduction in the prevalence of ARI from 13% in 1996 to 4% in 2014. These findings are in agreement with global efforts to reduce under five mortality and efforts to end child deaths due to ARI and diarrhoea [1, 4]. The reduction that was noted in the prevalence of ARI can be attributed to the efforts by the Zambian government through the introduction of the haemophilus influenza vaccine (HibHepB) in 2005 and the pneumococcal conjugate vaccine (PCV10) in 2013 [11, 13]. The findings of our study are consistent with the study conducted in Nigeria which saw a reduction in the prevalence of ARI related to the introduction of new vaccines to the routine EPI schedule [14]. Analysis of trends by residence showed a higher prevalence in the rural area as compared to urban areas. Similar findings were seen in a study conducted in Ghana which showed a higher prevalence of ARI in the rural area [15]. This finding can be related to the poor socioeconomic status associated with rural areas including overcrowded homes, mostly poor ventilation and use of unclean fuels. The opposite was noted in 2007 ZDHS were the prevalence was higher in urban areas. However, a contrary finding was observed in the 2007 ZDHS showed a higher prevalence in the urban areas [16].

Similar results were seen from a study conducted in India which showed a higher prevalence in the urban areas [17]. The reason for this finding could be related to overcrowding that occurs in some urban settings as a result of people moving from rural to urban areas. Although vaccination plays a major role in disease prevention, it is worth noting that our study findings did not show statistically significant association between ARI morbidity and vaccination at multivariable analysis. However this was not a finding unique to this study as studies conducted in India and a meta-analysis conducted in sub-Saharan Africa have shown a reduction in the prevalence of ARI with the introduction of the PCV vaccine [5, 17, 18]. Among sociodemographic variables associated with ARI, mother´s age, education level and child´s age were associated with ARI. Maternal age was associated with reduced odds for developing ARI. This result is in line with findings from studies conducted in Brazil, Ghana, Nigeria and Tanzania which showed that children born to mothers younger than 20 years had higher odds for developing ARI compared to mothers older than 20 years [8, 15, 19, 20]. This finding can be related to the inexperience, immaturity and unpreparedness among these adolescent mothers. On the contrary, studies conducted in different parts of Ethiopia found no significant associations between mother´s age and occurrence of ARI among under five children [5, 21].

Children who were under one year old were more likely to have ARI compared to older children. The finding is consistent with studies conducted in Rwanda, Ethiopia, Ndola (Zambia) [2, 22, 23] which showed increased likelihood of ARI. The increase in the risk for ARI in this age group is most likely related to the low immunity among the children which tends to improve secondary to exposures to immunizations and generally developing resistance to infections. Children younger than 11 months may be at increased risk due to incomplete vaccinations such as PCV, DPT HibHepB and measles [19]. Contrary to our study findings as well as findings from most studies which show the most affected age group to be among the 0-11 months, a study conducted in Nigeria in 2014 found higher prevalence among children aged 10-19 months, an age which was consistent with high complementary feeding which also increases the risk of exposure to other risk factors associated with ARI [19]. ARI prevalence of 16% was noted among underweight children. This finding is consistent with studies done in Nigeria and Tanzania which equally showed a higher prevalence of ARI among children who were malnourished and of low weight for age [19, 20, 24, 25]. A study conducted at the University Teaching Hospital Zambia also showed high prevalence of ARI and diarrhoea among malnourished children [26]. This finding further underscores the effect of underweight and undernutrition on the occurrence of ARI. Underweight indicates long term malnutrition which weakens the body and increases risk for infections.

This finding is related to the low immunity of underweight children which increases their vulnerability to developing infections including acute respiratory infections [19]. Children who did not receive vitamin A in the last 6 months before the survey were more likely to have ARI. A study conducted by Ramezani *et al*. 2015, showed that vitamin A deficiency was associated with increased susceptibility to infection due to the changes that occur in the epithelial tissue of the lung tissue [27]. Our study showed that children with mothers having secondary or higher education were less likely to develop ARI. These findings are consistent with findings from a study conducted in Kenya, Ethiopia and Rwanda [21, 28]. This finding may be related to the fact that these mothers may have access to literature which helps them to protect their children. A study conducted in Uganda found no association between maternal education and occurrence of ARI [29]. Among variables related to housing and environmental, cooking fuel and associated with ARI. Cooking fuel was associated with increased odds for ARI, children from homes were charcoal and wood were used for cooking had increased odds for ARI as compared to those were electricity was used. These findings are similar to the findings from a multi-country study which was conducted in Africa showed that the use of charcoal coal and wood was associated with increased risk for acute respiratory infections and ARI [25, 30].

A number of studies conducted in various parts of Ethiopia have also shown increased risk for ARI among children from homes which use unclean cooking fuel such as charcoal and firewood [5, 21]. Studies conducted in India, Nepal, Rwanda and Nigeria also showed that cooking fuel such as wood and charcoal was associated with increased risk for ARI as compared to electricity [22, 24, 25, 31, 32]. These findings can be attributed to the environmental pollutants such as wood smoke and charcoal smoke which results in the production of respirable particulate matter which increases the risk for respiratory infections such as ARI in children below the age of five years [8, 19, 22, 24, 33]. Wood smoke and charcoal smoke also reduces respiratory clearance functions and increases risk for infections [32]. Our study utilized secondary data from four different surveys as such the effect of some variables noted in literature were not assessed for example, the effect of wealth index, type of roofing material. Secondly, the outcome variable ARI was based on self-report from the mothers, possible recall bias. The newest survey was at least four years at the time of the analysis. However, the study was able to chat the trends and identify the factors associated with ARI among under five children and it is hoped that these findings will help the ministry of health child health unit as well as health promotion and social determinants to plan interventions which will contribute to the reduction of ARI. The findings of the study are generalizable since we utilised nationally representative data.

## Conclusion

A downward trend has been observed in the prevalence of ARI among under five children in Zambia. Young age, being underweight, use of cooking fuels such as wood and charcoal have been associated with increased risk for ARI. Having vitamin A supplementation, maternal education and have been found to be protective against ARI among children. There is need to introduce/scale up nutrition programs focusing on reducing malnutrition among under five children and introduction of health promotion programs to address overcrowding and use of unclean cooking fuels such as charcoal and firewood.

### What is known about this topic

- The prevalence of ARI among under five children in Zambia;
- The predisposing factors to ARI at global level and strategies to end child death due to pneumonia and diarrhoea.

### What this study adds

- The trends of ARI among children in Zambia;
- Using nationally representative data factors associated with ARI among under five children are identified and include biomass fuel and poor nutrition which are more common in the rural areas;
- Micronutrient supplementation such as vitamin A has been found to be protective hence it should be emphasized at all levels of healthcare.
